# Clinical features and treatment outcome of wrist tuberculosis in adult- a retrospective study of 84 consecutive cases with minimum of 2 years follow up

**DOI:** 10.1186/s12891-022-05563-8

**Published:** 2022-06-28

**Authors:** Maimaiaili Yushan, Ainizier Yalikun, Yimurang Hamiti, Cheng Lu, Aihemaitijiang Yusufu

**Affiliations:** Department of Microrepair and Reconstruction, The First Affiliated Hospital of Xinjiang Medical University, Urumqi, Xinjiang China

**Keywords:** Antitubercular chemotherapy (ATT), Surgical treatment, Tuberculosis, Wrist

## Abstract

**Background:**

Wrist tuberculosis (TB) is a rare disease that may result in residual deformity, pain, or stiffness even after proper antitubercular chemotherapy (ATT) and surgical intervention. The aim of our study is to present clinical features and functional outcomes of wrist TB in a consecutive series of 84 adult patients with a minimum of 2 years of follow-up.

**Methods:**

Clinical features and treatment outcomes of 84 consecutive adult patients with wrist TB from January 2003 to June 2018 including 45 men and 39 women, with a mean age of 46.8 years (18–84) were retrospectively analyzed. Data were collected on participants’ demographic details. The primary outcome measures were QuickDASH score, grip strength, Visual Analogue Scale (VAS), and PRWHE. Secondary outcomes include health-related quality of life was evaluated using the EuroQol five-dimension five-level (EQ-5D-5L), assessment and postoperative complications of patients who underwent operation were also recorded.

**Results:**

All 84 patients with an average follow-up of 50.8 (24–105) months. The mean duration of symptoms before treatment was 10.5 months (2–21). There were 27 patients with bony and 57 with primarily soft-tissue involvement based on preoperative evaluation of plain radiographs and MRI. There were 33 patients treated with ATT and 51 patients were treated with surgery followed by ATT. Among them, 13 patients (15.5%) underwent incision and decompression, 14 patients (16.7%) underwent wrist synovectomy, 13 patients (15.5%) underwent wrist joint fusion by plate fixation, and 11 patients (13.1%) underwent wrist joint fusion by external fixation. At the last clinical visit, the QuickDASH, and PRWHE scores of all patients decreased significantly, the VAS improved from 5.9 to 1.4, EQ-5D-5L utility index improved from 0.36 to 0.88, EQ-VAS improved from 40.2 to 89.1. All patients indicated good wrist recovery at the last follow-up, and the treatment achieved satisfactory clinical outcomes.

**Conclusion:**

The onset of wrist TB is insidious; early diagnosis, good patient compliance, and surgery combined with ATT are crucial steps for treatment of wrist TB, and also essential for the patient's postoperative recovery. Wrist arthrodesis has achieved satisfactory results in the treatment of severe wrist TB.

**Supplementary Information:**

The online version contains supplementary material available at 10.1186/s12891-022-05563-8.

## Introduction

Tuberculosis (TB) has been documented as far back as Egyptian mummies [1]. In developed countries, the presence of immunodeficiency and the emergence of drug resistance poses fresh challenges. On the contrary, the natural history of the disease with a sizable number of patients seeking medical treatment the first time in advanced stages have been continually reported in the developing country [2]. No patient of any age or sex is immune from skeletal TB and treatment outcome has been dramatically improved since the introduction of antitubercular therapy (ATT). Musculoskeletal manifestations have remained the most common form of extrapulmonary tuberculosis, reaching up to 10% of these cases [3]. Hand and wrist TB is a rare medical condition that accounts for less than 1% of all skeletal TB. Due to the sophisticated anatomical structure of the wrist joint and non-specific or atypical clinical features, timely diagnosis and proper treatment is still a challenging situation for orthopedic surgeons.

Wrist TB is a paucibacillary disease defined as a chronic infection caused by *Mycobacterium tuberculosis* which involved distal radius/ulna, carpal bone, metacarpal joints, and surrounding soft tissue including synovium, ligaments, flexor and extensor. Clinical features are usually atypical such as wrist swelling, intermittent pain, restricted motion, digital numbness caused by median nerve involvement, or draining sinus and joint stiffness in advanced cases. Diagnosis of wrist TB only can be established through a combination of past medical history, current clinical and imaging evaluation, sample examination including mycobacterial culture, acid-fast bacilli smear, histological and/or cytological test, and molecular methods. Progressive invasion to the surrounding soft tissue may cause residual deformity, pain or stiffness even after proper ATT and surgical intervention if delayed in diagnosis. Surgery is indicated for a patient with an established diagnosis who is not responsive to a standard ATT for 8 weeks, or overt radiographic sequestrum with large obsess formation and compromised hand function. Patient who suffered severe wrist joint dysfunction even with effective ATT is also indicated for operation to achieve better functional recovery.

Ely LW [4] firstly reported surgical treatment of wrist TB in 1920, two cases of wrist TB were successfully treated by radical debridement and spontaneous wrist arthrodesis with “rod” shaped tibial grafting and postoperative immobilization using plaster of Paris. Due to its rarity, studies on the treatment outcome have been rarely reported [5–10]. Surgical treatment is varied based on the involvement of bone, joint, and synovium, severity of the bony destruction, influence of hand/wrist function, and the effect on the patient’s daily life, which include radical debridement of the lesion with or without bone grafting and immobilization using casting, plate fixation or external fixation. This study aimed to present clinical features and functional outcomes of wrist TB in a consecutive series of 84 adult patients with a minimum of 2 years of follow-up.

## Methods

### Patients

This is a retrospective observational study and approved by the Ethics Committee of the First Affiliated Hospital of Xinjiang Medical University. In this study, adult patients with wrist TB who underwent either conservative therapy (ATT) or surgical intervention from January 2003 to June 2018 in our institution with a minimum of 2 years of follow-up were enrolled. Inclusion criteria: aged above 18 years; confirmed diagnosis of wrist mycobacterium TB infection which was established by a core biopsy or fine-needle aspiration depending on involvement before operation. Patients were excluded who were under 18 years old, had TB involvement of other musculoskeletal systems, previous medical history of operative treatment, and were unwilling to participate or lost of follow-up. Detailed general information includes sex, age, side of infection, location, course duration prior to the operation, chief complaint, and clinical manifestation. Radiographs of the affected wrist and chest were taken to evaluate lesion involvement and to exclude healed or currently active pulmonary TB. Patients with suspected soft tissue involvement on a plain radiograph was further evaluated by MRI. Epitrochlear and axillary lymph node examination was performed routinely. Patients with obvious radiological lesions were scheduled for core biopsy or fine-needle aspiration cytology (FNAC) under image guidance and obtained samples sent for acid-fast stain and culture on Lowenstein-Jensen medium, culture and sensitivity, gram stain, and histopathological study. A standard ATT (rifampicin 10 mg/kg, isoniazid 5 mg/kg, pyrazinamide 20 mg/kg, and ethambutol 15 mg/kg per day) was administered for 2 weeks in the waiting period of the culture results, then adjusted based on the sensitivity of the culture results and followed by12-18 months of ATT once histological results were confirmed with tubercular granuloma. Patients who were not responsive to a standard or adjusted ATT at least for 8 weeks or demonstrated obvious radiographic sequestrum with large obsess formation and compromised hand/wrist function were scheduled for operation and informed consent was acquired. Prophylactic cefuroxime was administered 3 days before the operation to eliminate bacteria and prevent infection. A thorough nerve examination should be performed to rule out the need for simultaneous nerve decompression at the time of operation. All patients included in this study were operated on by the same surgical team. Demographic details of included patients are shown in Table 1.

**Table1:** Demographic data and clinical characteristics

Age, years	46.8 ± 18.5
Gender	
Male,n(%)	45(53.6%)
Female,n(%)	39(46.4%)
Side of infection	
Left wrist,n(%)	32(38.1%)
Right wrist,n(%)	52(61.9%)
Dominant wrist,n(%)	60(71.4%)
Duration of symptoms,months,n(%)	10.5 ± 3.3
Follow-up, months	50.8 ± 17.1
History of pulmonary tuberculosis,n(%)	9(10.7%)
History of wrist trauma,n(%)	24(28.6%)
Bony involvement,n(%)	27(32.1%)
Capitate,n(%)	7(8.3%)
Lunate,n(%)	5(6.0%)
Scaphoid,n(%)	4(4.8%)
Ulna,n(%)	6(7.1%)
Radius,n(%)	5(6.0%)
Soft-tissue involvement,n(%)	57(67.9%)
Tuberculous tenosynovitis,n(%)	38(45.2%)
wrist synovitis,n(%)	19(22.6%)
Treatment(surgical),n(%)	51(60.7%)
Wrist arthrodesis (internal fixation),n(%)	13(15.5%)
Wrist arthrodesis (external fixation),n(%)	11(13.1%)
Synovectomy,n(%)	14(16.7%)
Incision and drainage,n(%)	13(15.5%)
Treatment(conservative),n(%)	33(39.3%)

### Surgical procedure

The patient was placed in the supine position with the arm extended and hand pronated on a hand table under brachial plexus block or general anesthesia. A tourniquet is employed in all cases for a better exploration of the surgical field. A dorsal or palmar “S” shaped longitudinal incision is made in line with the long finger depending on the preoperative evaluation of the lesion involvement. In patients with massive bony destruction or severe deformity, the incision should be carefully designed to follow a line from the middle finger metacarpal base through the lunocapitate joint and across the lunate fossa of the radius for better visualization and mobilization of tissues. Patients with soft tissue (synovium and peritendineum) involvement underwent radical debridement and lesion excision, transverse carpal ligaments were opened, and the wrist tendon was carefully separated from the transverse ligament of the flexor/extensor carpi. Patients with carpal bony destruction underwent radical removal of the necrotic bone and repeated curettage followed by wrist arthrodesis and the wrist was fixed in a functional position using plate or external fixation, bone grafting is necessary in cases with inadequate wrist stabilization for better functional restoration. The autologous bone was harvested from the Iliac crest for bone grafting. Taken samples were sent for general bacterial culture, bacterial and fungal smear, acid-fast staining, and histopathological study. Wound was flushed repeatedly with normal saline and a drainage was the place for postoperative streptomycin irrigation for 3 to 4 weeks which could reduce local bacterial reproduction and be beneficial to the incision healing.

### Postoperative management

Patients were allowed for the active and passive exercise of metacarpophalangeal and interphalangeal joints initiated on day 1 after the operation. Wrist swelling, drainage and general nutritional status were closely monitored. All patients were administered with a standard ATT (rifampicin 10 mg/kg, isoniazid 5 mg/kg, pyrazinamide 20 mg/kg, and ethambutol 15 mg/kg per day) for 3 months and changed to ATT of two drugs (rifampicin 10 mg/kg, isoniazid 5 mg/kg) for the following 9 months. Patients were followed-up monthly in the first six months, and every two months from six months to one year, and then every six months in the second year. Hepatic and renal function, Erythrocyte Sedimentation rate (ESR), and C-reactive protein (CRP) were regularly monitored during each visit until their value returned to the normal level. Radiographic evaluation during each visit is required. Additional examination such as CT or MRI has considered if the patient suffered unexpected pain or any other uncomfortable symptoms during the follow-up period. Regular dressing of the pin tract was applied for patients who underwent external fixation. The external frame is removed when there is an occurrence of fibrotic adhesions or callus formation around the wrist joint to obtain fibrous ankylosis of the joint and stabilization of the local structure.

### Outcome measurement

All included patients were followed up for a minimum of 2 years in accordance with postoperative follow-up appointments. The primary outcomes were measured using a visual analog scale (VAS: 0 to 10, with 10 representing intolerable pain) at the last visit where patients rated their pain on the movement of radial and ulnar deviation both at rest and during activity. They were asked to complete the form Quick Disabilities of Arm, Shoulder, and Hand (QuickDASH) score [11] and Patient-Related Wrist and Hand Evaluation (PRWHE) [12]. Grip strengths were measured using a Jamar dynamometer and compared with the contralateral side. Secondary outcomes were measured by the EuroQol five-dimension five-level (EQ-5D-5L) [13] assessment which is a standardized scoring system for evaluating generic health status, the maximum score of the EQ-5D-5L index is 1, and a lower score indicates worse health status. Patients who underwent operation were closely followed up for postoperative complications which were recorded and managed properly. Regular radiographic evaluation was ordered and compared with the previous one to monitor treatment effectiveness and identify possible recurrence.

### Statistical analysis

All statistical analyses were conducted using SPSS Statistical Software (SPSS for Windows, version 25.0). Continuous variables were compared by using a t-test, and data are presented as the mean and standard deviation. A *p*-value of < 0.05 was considered significant.

## Results

A total of 84 patients were enrolled in this study. There were 45 males (53.6%) and 39 females (46.4%) with an average age of 46.8 (18–84) years and an average follow-up was 50.8 (24–105) months. The mean duration of symptoms before treatment was 10.5 months (2–21). There were 27 patients (32.1%) with bony and 57 (67.9%) with primarily soft-tissue involvement based on preoperative evaluation of plain radiographs and MRI. Among them, 7 (8.3%) cases involved capitate, 5 cases (6.0%)involved lunate, 4 (4.8%)cases involved scaphoid, 6 cases (7.1%) involved distal ulna, 5 (6.0%)cases involved distal radius, 38 cases (45.2%)involved wrist tenosynovium (especially the flexor side of wrist) and 19 (22.6%) cases involved synovium of the wrist joint. After a detailed medical history inquiry, 9 patients (10.7%) had a previous history of pulmonary TB, 24 patients (28.6%) had a previous history of wrist trauma, 60 patients (71.4%) had dominant hand involvement, and no active pulmonary TB was found in all patients.

Pain and swelling were the most common clinical manifestations, and other clinical manifestations were wrist mass, numbness, stiffness, and limited mobility, and there were 15 patients who had wrist ulceration and exudation. There were five patients had symptoms of median nerve entrapment at the wrist, and the results of the electromyogram(EMG) suggested carpal tunnel syndrome(CTS). Three of them were tuberculous tenosynovitis of the wrist, which improved after incision and drainage, and two were wrist synovitis, which improved after synovectomy. There were 33 patients treated with ATT and 51 patients were treated with surgical treatment combined with ATT. Among them, 13 patients (15.5%) underwent incision and decompression, 14 patients (16.7%) underwent wrist synovectomy, 13 patients (15.5%) underwent wrist joint fusion by plate fixation, and 11 patients (13.1%) underwent wrist joint fusion by external fixation. All patients continued ATT after surgical treatment. Intraoperative secretion culture of the wrist did not reveal any resistant bacteria, and "rice bodies" were found in the wrist tissues of 8 patients (9.5%). In our study, one patient who underwent wrist joint fusion by plate fixation and another patient who underwent incision and drainage had recurrence at 10 months and 11.5 months after the operation, respectively, and who were managed by radical debridement + continuous ATT. The ESR and CRP of the other patients returned to normal after ATT.

There were five patients who had symptoms of median nerve entrapment at the wrist in our study, and the results of the electromyogram(EMG) suggested carpal tunnel syndrome(CTS). The intraoperative observations showed that the median nerve and all the flexor tendons were surrounded by thick synovial tissue, and the median nerve under the flexor retinaculum was flattened. Median nerve and flexors were released intraoperatively, and the numbness symptoms of the five patients recovered after the operation.

**Fig. 1:**
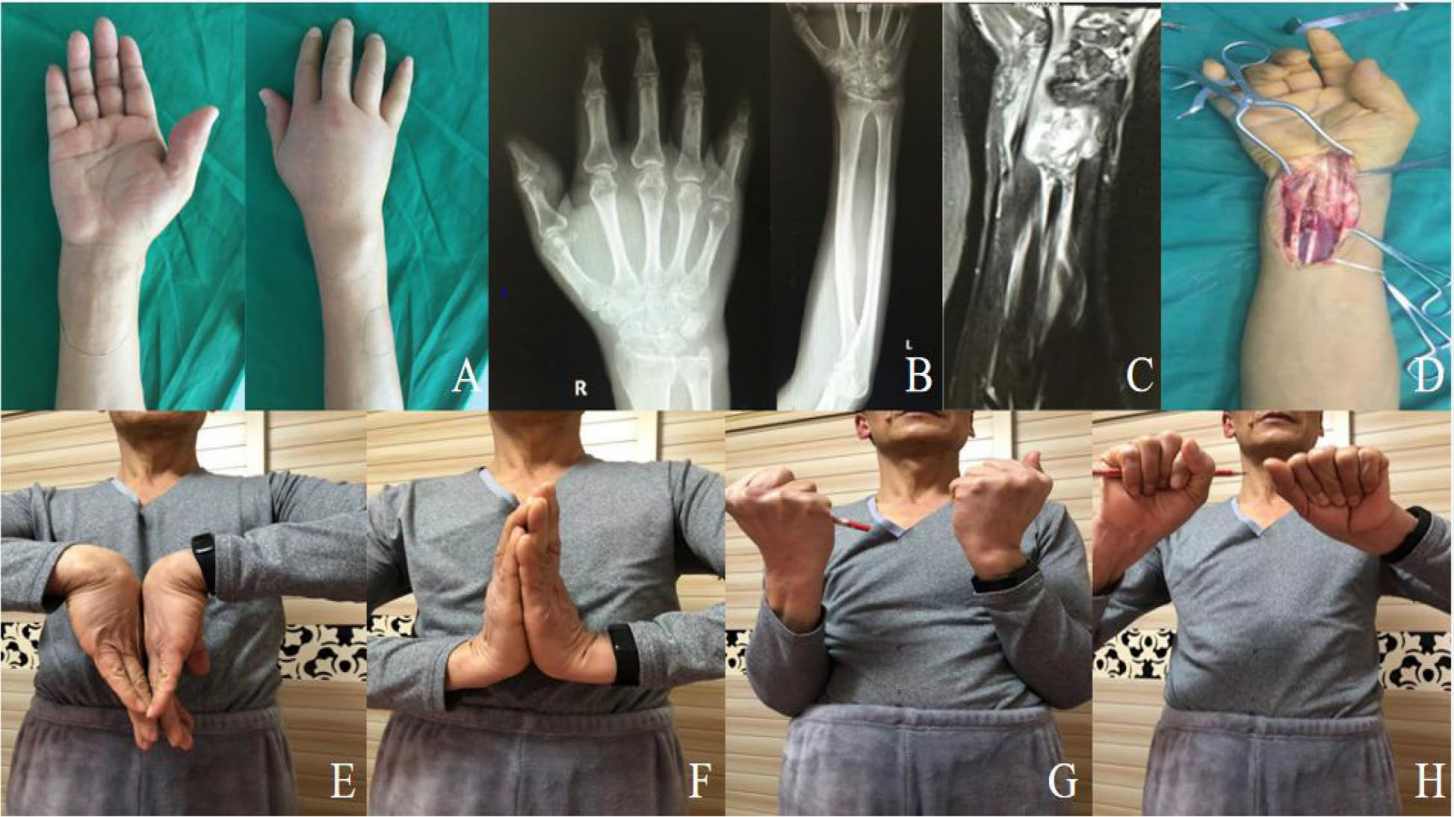
A 52-year-old male patient with Tuberculous tenosynovitis of the wrist. **A**. Swelling can be observed on the palmar and dorsal side of the right wrist. **B**. No obvious bone destruction was found on X-ray films of the wrist joint and distal ulnar and radius. **C**.MRI showed that the synovial lesions of the wrist involve the flexor tendons and the surrounding soft tissue. **D**. An intraoperative photograph suggested that the degenerated and necrotic synovial tissue attached around the flexor tendons had been completely removed. **E**–**H**. The patient's right wrist function recovered well 25.5 months after the operation

**Fig. 2:**
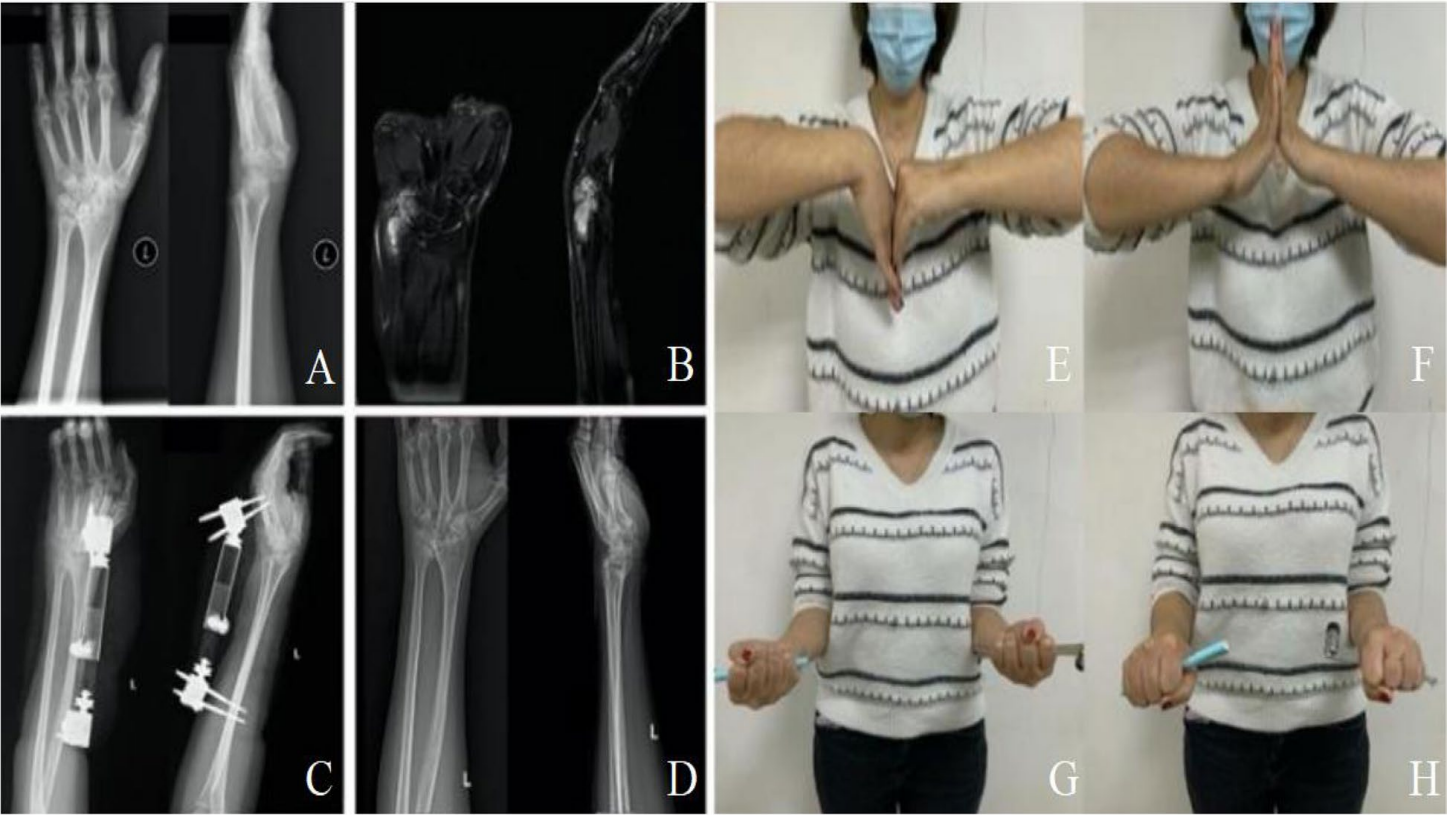
A 43-year-old female patient with Wrist Tuberculosis. **A**. Anteroposterior and lateral X-rays reveal an osteolytic lesion of the distal radius and the proximal carpal bones. **B**. MRI images of the left hand showed destructive lesions in the distal ulna and carpal bones, surrounding local effusion, and osteolytic lesions with stenosis of the intercarpal and ulna-carpal joint. **C**. Anteroposterior and lateral X-rays show 2 days after wrist joint fusion by external fixation. **D**. The X-ray revealed wrist status 12 months after the removal of external fixation. (**E**–**H**). 27 months after the removal of the external fixation, the patient's left wrist function recovered well

**Fig. 3:**
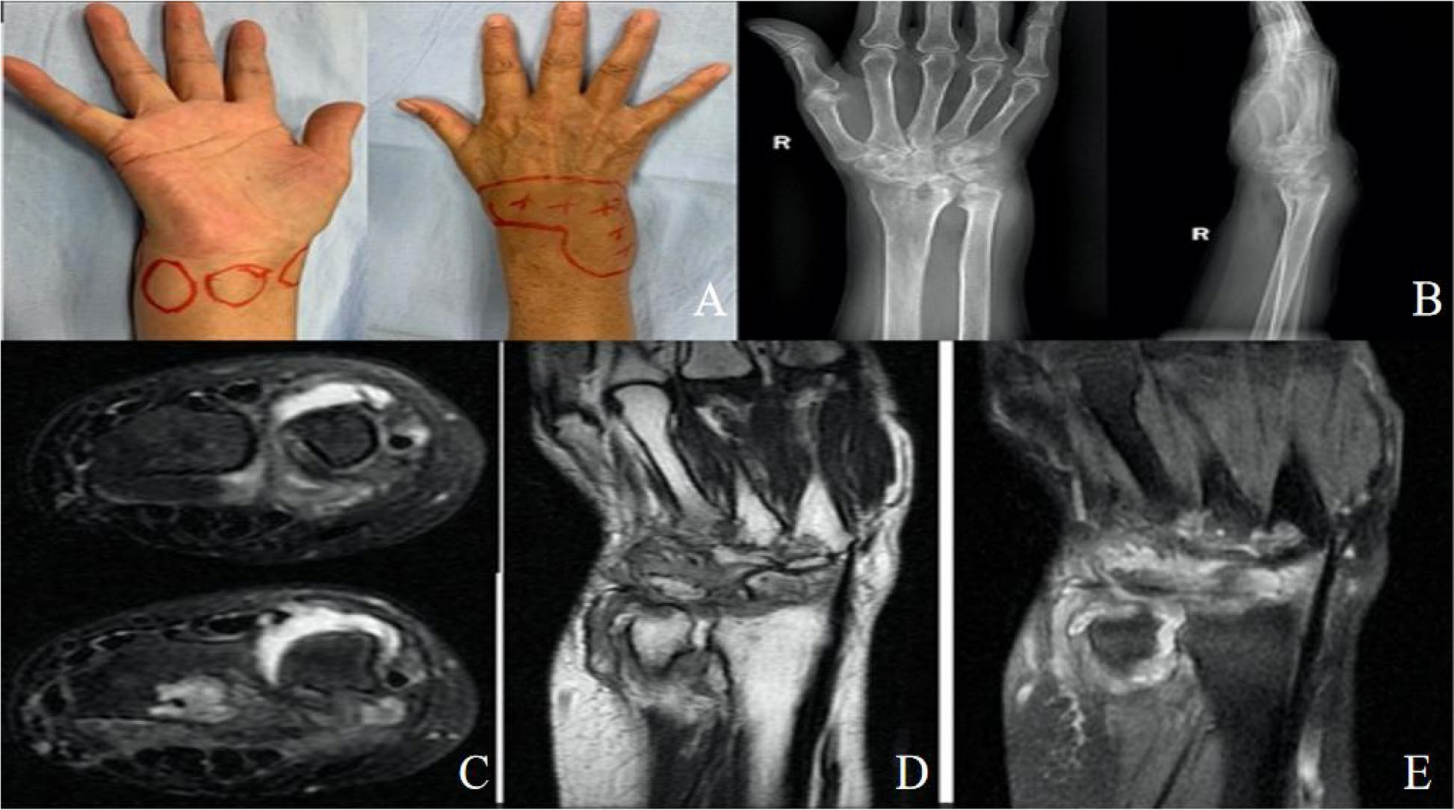
A 49-year-old male patient who received conservative treatment. **A**. Swelling can be observed on the palmar and dorsal side of the right wrist. **B**. Anteroposterior and lateral X-ray of the right wrist revealed malalignment of the wrist with an osteolytic lesion of the distal radius, distal ulna, and the proximal carpal bones. **C**. MRI images of the right hand showed osteolytic lesions in the distal ulna and carpal bones, and extensive necrotic degeneration of the synovium and surrounding soft tissues of the wrist joint

**Fig. 4:**
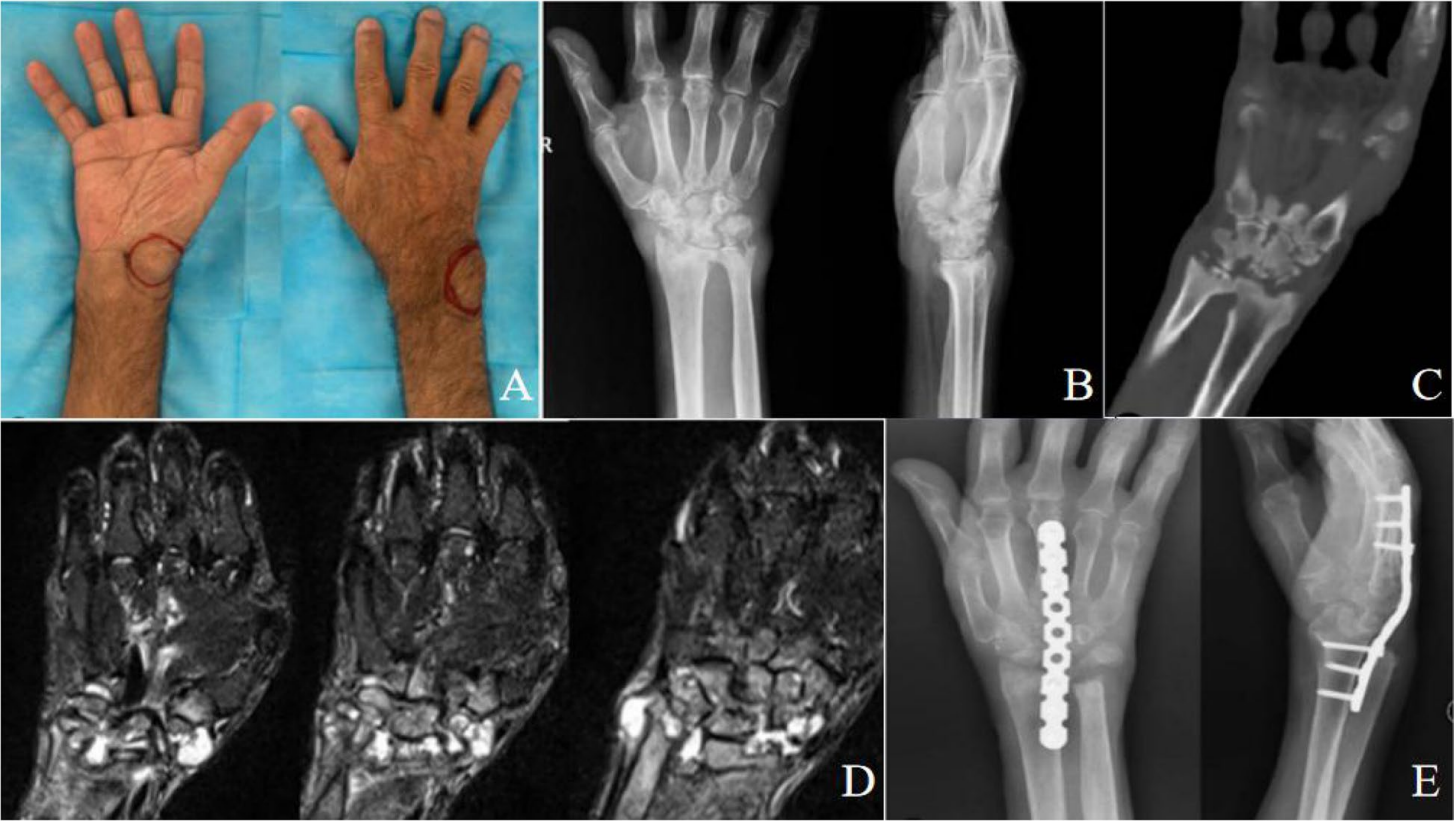
A 46-year-old male patient with wrist tuberculous. **A**. Swelling can be observed on the palmar and dorsal side of the right wrist. **B**,**C**. Anteroposterior, lateral X-ray, and CT scan of the right wrist showed an osteolytic lesion of the distal radius, distal ulna, and carpal bones. **D**, MRI images of the right hand showed significant osteolytic lesions in the distal ulna and carpal bones, and extensive necrotic degeneration of the synovium of the wrist joint. **E**, Anteroposterior and lateral X-rays showed 28 months after wrist joint fusion by plate fixation

The QuickDASH, PRWHE, VAS, EQ-5D-5L utility index, and EQ-VAS scores of all patients before and after the operation were statistically analyzed, and the difference was statistically significant (see Table 2). The wrist function and grip strength of all patients were remarkably improved. Grip strength increased from preoperative 12.5 kg to 32.4 kg, from 32.9% to 85.5% of contralateral grip strength; the improvement of VAS was observed in all patients, from the preoperative 5.9 to 1.4. All 15 patients with wrist ulceration and exudation underwent surgical treatment, and patients in the surgical group had more severe infections involving bone or deep soft tissues. At the last follow-up, the EQ-VAS scores of patients in the surgical group and the nonsurgical group were 88.0 and 90.3, respectively. All patients indicated good wrist recovery at the last follow-up, and the treatment described above achieved satisfactory clinical outcomes. (Typical cases see Figs. 1, 2, 3 and 4).

**Table 2:** Preoperative and postoperative functional evaluation(Valued at last follow-up)

	Preoperative	Postoperative	*p*-value
QuickDASH	55.4 ± 8.5	8.0 ± 2.8	< 0.001
PRWHE	59.4 ± 11.0	9.5 ± 2.6	< 0.001
EQ-5D-5L utility index	0.36 ± 0.18	0.88 ± 0.12	< 0.001
EQ-VAS	40.2 ± 12.5	89.1 ± 8.4	< 0.001
Grip strength, kg	12.5 ± 3.1	32.4 ± 4.8	< 0.001
Grip Strength, (% Contralateral Side)	32.9 ± 6.9	85.5 ± 5.0	< 0.001
VAS	5.9 ± 1.6	1.4 ± 0.5	< 0.001

*QuickDASH* Quick Disabilities of Arm, Shoulder and Hand score

*PRWHE* Patient-Related Wrist and Hand Evalution score

*EQ-5D-5L* EuroQol five-dimension five-level

*VAS* visual analog scale/score

## Discussion

Wrist TB is a specific medical condition that usually affects the adult population, there are no gender differences and only one side was involved in most cases. It’s caused by secondary infection- TB bacilli, which spread to the wrist through blood circulation or lymph node in most cases, and result in swelling, pain, deformities, or even hand and wrist dysfunction in the end-stage if delay in diagnosis. Tendon sheaths were involved in most cases with “cold” abscess formation which penetrated into the surrounding structure (joint capsule, muscle, tendon, and synovium) through interstitial spaces resulting in synovium thickening, coagulated granuloma, necrotic tissue followed by tendon adhesion and functional impairment. Complete resection of the infected lesion and functional reconstruction is still a challenge to orthopedic surgeons considering the complex and sophisticated anatomical structure of the wrist, and unrealistic expectations in advanced cases. Some patients may suffer non-healable discharging sinus, bone, and tendon exposure or defects, limb deformities, and shortening after the operation, which may result in temporary or permanent hand and wrist deformities and dysfunction. How to effectively control the infection and to achieve the functional recovery, prevention of the postoperative complications and occurrence of residual deformities is a challenging issue.

Tuberculous infection of the musculoskeletal system comprises 10% of all extrapulmonary cases of TB [14]. Although musculoskeletal TB frequently affects the spine (51%), pelvis (12%), hip and femur (10%), knee and tibia (10%), and ribs (7%) [15], tuberculous involvement of the wrist is rare, less than 1% [16, 17]. Watts et al. [15] proposed that approximately one-third of musculoskeletal TB involed other organ such as the lung involvement. Chao et al. [18] reported 8 cases of wrist TB, of which 1 case (12.5%) was found to have active pulmonary TB. There were no patients with active pulmonary TB or extrapulmonary TB but the wrist in our study, which was the same as the study by Kotwal and Khan [10], but 9 of our cases had a previous history of pulmonary TB. Several reports pointed to an association between mechanical factors such as trauma and the development of musculoskeletal TB. Yao et al. [19] demonstrated that in musculoskeletal TB, previous history of trauma is common, with a proportion of up to 30% – 50%. A study reported that 30 of 99 patients with musculoskeletal TB had a history of previous trauma, they indicated trauma may be associated with musculoskeletal TB because of resulting increased vascularity, decreased resistance, or unmasking of latent infection [20]. Hassanpour et al. [21] noted that joint trauma and over use of the wrist joint are important predisposing factors for musculoskeletal TB. In this study, 24 patients (28.6%) had a previous history of wrist trauma, and 60 patients (71.4%) were involved in the dominant hand, which may further explain the above arguments.

The X-ray findings of wrist TB are diverse, including bone cysts, osteolytic lesions (the osteolytic findings of TB are often surrounded by sclerotic margins), reactive sclerosis, localized osteoporosis, joint destruction, and periosteal reaction, which can erode the epiphysis in children [22]. These findings are not specific and may deceptively mimic inflammatory arthritis, pigmented villonodular synovitis, gout, or soft-tissue tumor [23]. A classic triad of radiological findings, known as Phemister’s triad, which includes the presence of juxta-articular osteoporosis, peripheral bony erosions, and gradual joint space narrowing suggests the presence of tubercular arthritis [24]. The joint space is preserved early in tuberculous arthritis, as opposed to most pyogenic infections, in which there is the early destruction of cartilage due to the production of proteolytic enzymes by bacterial pathogens [20]. More notably, in some cases, local pain, swelling, and limitation of motion may sometimes appear even 8 weeks earlier than radiological examination [25]. Therefore, MRI is the preferred imaging examination for musculoskeletal TB. MRI can detect earlier changes, especially synovial thickening and periarticular soft tissue changes, and has even been shown to demonstrate “rice bodies” in the joint space [26]. MRI may demonstrate hemosiderin deposition in the synovium and demonstrate erosions that occur late in the disease and are usually centrally located. The absence of bone marrow enhancement on MRI accompanied by bone erosion suggests tuberculous arthritis rather than suppurative lesions [27].

“Rice bodies” were first described in German literature in 1895, and later in the English literature in 1927 by Rogers [28]. The etiology of rice bodies is still subject to debate. They are thought to arise from chronic bursitis, whereas some authors suggest that they may arise from microinfarctions with subsequent synovial shedding and encasement by fibrin [29]. Some reports have noted the presence of rice bodies in less than 50% of cases [30]." Rice bodies" were found in the wrist tissues of 8 patients (9.5%) in our study. Woon et al. emphasized [28] that intraoperative findings of "rice bodies" are highly suggestive of tuberculous tenosynovitis. However, it should be pointed out that “rice bodies” can also be seen in other wrist diseases, such as seronegative arthritis, rheumatoid arthritis, systemic lupus erythematosus (SLE), and osteoarthritis of the joint [31]. Hassanpour reported [21] the clinical characteristics of 12 patients with Mycobacterium tuberculosis–induced CTS, they believed that there may be no correlation between TB-induced CTS and pulmonary TB based on clinical signs and symptoms or radiologic findings.

The mean duration of symptoms before ATT was 10.5 months in the present study, and some patients had a more severe infection. In addition, the onset of wrist tuberculosis is insidious, and it is difficult to distinguish it from malignant tumors, chronic infections, and other diseases on imaging, which brings difficulties to the diagnosis, so it is often missed and misdiagnosed, and the disability rate is high [32]. Bayram et al. [33] emphasized that wrist TB takes an average of 16–19 months to diagnose because early diagnosis is difficult, and the misdiagnosis rate is high. Therefore, early diagnosis and treatment of wrist TB are essential for the recovery of joint function.

Of 51 surgical patients, 15 patients with wrist ulceration and exudation and 3 patients with median nerve entrapment underwent surgery directly after 2 weeks of ATT. The remaining 33 patients had no significant improvement and underwent surgery after 8 weeks of ATT. Many scholars believed that in order to prevent bone destruction and disease dissemination, preoperative ATT is essential [34, 35]. Two patients had a recurrence of infection 10 months and 11.5 months after the operation, including one patient with a history of multiple wrist steroid injections and the other patient who stopped ATT 3 months after the operation. The remaining surgical patients recovered after 12–18 months of regular ATT after surgery. Chandrasekharan et al. [36] concluded that irregular postoperative administration of ATT and a history of joint steroid injection may be factors leading to wrist tuberculosis recurrence, which is consistent with our study.There were significant differences in QuickDASH, PRWHE, and EQ-5D-5L utility index scores between surgical and non-surgical patients. (See Table 3) We considered this not to mean that the effect of conservative treatment is better, because the symptoms of the operation group and non-operation group last 11.4 months and 9.0 months respectively, surgical patients are more likely to miss the optimal treatment time due to delayed diagnosis or misdiagnosis, resulting in a more serious condition.

**Table 3:** Comparison of Nonsurgical and Surgical group (Valued at last follow-up)

	Nonsurgical(*n* = 33)	Surgical(*n* = 51)	*p*-value
Age, years	44.2 ± 19.6	48.5 ± 17.8	0.298
Duration of symptoms,months	9.0 ± 2.9	11.4 ± 3.2	0.001
QuickDASH	6.6 ± 2.2	8.9 ± 2.8	< 0.001
PRWHE	7.9 ± 2.2	10.6 ± 2.3	< 0.001
EQ-5D-5L utility index	0.92 ± 0.10	0.85 ± 0.13	0.020
EQ-VAS	90.3 ± 7.4	88.0 ± 8.9	0.302
Grip strength, kg	33.7 ± 4.9	31.5 ± 4.5	0.037
Grip Strength, (% Contralateral Side)	86.1 ± 5.9	85.1 ± 4.3	0.333
VAS	1.1 ± 0.4	1.6 ± 0.5	< 0.001

Complete resection of infected tissue, including necrotic carpal bones, and tendons, especially caseous necrotic tissue and “rice bodies”, plays an important role in postoperative recovery of the wrist joint. Arthrodesis is feasible when the carpal bone or distal radius and ulna are severely eroded and wrist function cannot be recovered after resection of necrotic lesions. We performed wrist arthrodesis in 24 patients, 13 with internal fixation plates and 11 with external fixations. External fixation has been removed an average of 12.5 weeks after surgery. Ha et al. [37] insisted that M. tuberculosis rarely adheres to metal surfaces and forms little biofilm compared to Staphylococcus epidermidis. The follow-up results show that, after the operation, the QuickDASH, and PRWHE scores of all patients decreased significantly, the VAS, EQ-5D-5L utility index, and EQ-VAS score increased remarkably, and the grip strength improved significantly. We supposed surgery combined with ATT is the key to our success in the treatment of wrist TB.

The limitations of this study include its single-center retrospective design, and the case number is relatively small to reach a strong conclusion because wrist TB is particularly rare. Time interval after surgical intervention for patient with wrist TB in the present study should be in six months, twelve months, two years, and at the last clinical visit, which could demonstrate the trajectory changes during follow up period. However, most participants in the present study lives in remote area or village, regular follow-up at different time-interval is not an available option. In addition, adjustment of the baseline values was considered but not conducted, considering the rarity of the wrist TB and the number of the participants, which would be a valuable point to improve our future study.

## Conclusion

The onset of wrist TB is insidious; early diagnosis, good patient compliance, and surgery combined with ATT are crucial steps for the treatment of wrist TB and are also essential for the patient's postoperative recovery. Wrist arthrodesis has achieved satisfactory results in the treatment of severe wrist TB.

## Supplementary Information

**Additional file 1. **

## Data Availability

All data generated or analyzed during this study are included in this published article.

## Abbreviations

TB: Wrist tuberculosis
ATT: Antitubercular chemotherapy
CTS: Carpal tunnel syndrome
FNAC: Fine-needle aspiration cytology
CRP: C-reactive protein
ESR: Erythrocyte sedimentation rate
VAS: Visual analog scale
QuickDASH: Quick Disabilities of Arm, Shoulder and Hand
PRWHE: Patient-Related Wrist and Hand Evaluation
EQ-5D-5L: EuroQol five-dimension five-level
EMG: Electromyogram
